# Evaluation of ambulance offload delay at a university hospital emergency department

**DOI:** 10.1186/1865-1380-6-15

**Published:** 2013-05-10

**Authors:** Derek R Cooney, Susan Wojcik, Naveen Seth, Corey Vasisko, Kevin Stimson

**Affiliations:** 1 EMS Medicine Fellowship, Department of Emergency Medicine, SUNY Upstate Medical University, 550 East Genesee / EMSTAT Center, Syracuse, NY, 13202, USA; 2 Department of Emergency Medicine, SUNY Upstate Medical University, 550 East Genesee / EMSTAT Center, Syracuse, NY, 13202, USA; 3 Department of Emergency Medicine, SUNY Upstate Medical University, 750 East Adams Street, Syracuse, NY, 13210, USA; 4 College of Medicine, SUNY Upstate Medical University, 1215 Weiskotten Hall, 766 Irving Avenue, Syracuse, NY, 13210, USA; 5 EMS & Disaster Medicine, Department of Emergency Medicine, SUNY Upstate Medical University, 550 East Genesee / EMSTAT Center, Syracuse, NY, 13202, USA

**Keywords:** Ambulance, Diversion, Dropoff delay, EMS, EMTALA, NEDOCS, Offload delay, Parking, Prehospital, Return to service, Turn around time, Wait time

## Abstract

**Background:**

Ambulance offload delay (AOD) has been recognized by the National Association of EMS Physicians (NAEMSP) as an important quality marker. AOD is the time between arrival of a patient by EMS and the time that the EMS crew has given report and moved the patient off of the EMS stretcher, allowing the EMS crew to begin the process of returning to service. The AOD represents a potential delay in patient care and a delay in the availability of an EMS crew and their ambulance for response to emergencies. This pilot study was designed to assess the AOD at a university hospital utilizing direct observation by trained research assistants.

**Findings:**

A convenience sample of 483 patients was observed during a 12-month period. Data were analyzed to determine the AOD overall and for four groups of National Emergency Department Overcrowding Scale (NEDOCS) score ranges. The AOD ranged from 0 min to 157 min with a median of 11 min. When data were grouped by NEDOCS score, there was a statistically significant difference in median AOD between the groups (*p* < 0.001), indicating the relationship between ED crowding and AOD.

**Conclusion:**

The median AOD was considered significant and raised concerns related to patient care and EMS system resource availability. The NEDOCS score had a positive correlation with AOD and should be further investigated as a potential marker for determining diversion status or for destination decision-making by EMS personnel.

## Findings

### Background

Ambulance offload delay (AOD) is the time between patient arrival via emergency medical services (EMS) to the Emergency Department (ED) and the time that the patient is completely out of the care of the EMS crew. For the EMS crew to be clear of their duty to their patient, a report must be given to the ED staff and the patient must be moved off of the EMS stretcher. International attention is now being paid to this new marker of ED performance among hospital administrators and EMS system officials [1,2]. The National Association of EMS Physicians has released a position paper on the topic [3], and there is an accompanying resource document [4] that notes that AOD is thought to have a potentially greater impact on patient safety than ambulance diversion and return to service times. The study of AOD should provide a more accurate depiction of the true effect of ED crowding on ambulance availability. Concern over AOD was expressed in a communication from the United States Department of Health and Human Services [5] in a letter implying that “parking” of EMS patients in the hallway of an ED constitutes a violation of Emergency Medical Treatment and Labor Act (EMTALA) [6]. At the time of the initiation of this study, 30 min was thought to be an important benchmark for AOD because of the Canadian standard for ambulance offload time [7]. The objective of this pilot study was to evaluate the AOD at a busy university hospital ED, utilizing a method for direct observation of offload events.

### Hypotheses

The hypotheses in place at the start of the pilot study were: (1) The median AOD would be less than the Canadian standard of 30 min. (2) The median AOD would be longer when there is more ED crowding, as measured by the National Emergency Department Overcrowding Scale (NEDOCS) score.

## Methods

This study was an observational study of a convenience sample of 483 patients arriving via EMS to a level 1 academic trauma center during a 12-month period from 29 March 2010 through 28 March 2011. Trained research assistants (RA) directly observed EMS patient arrival, time of EMS report, and time of movement of the patient off of the EMS stretcher. At the time of EMS patient arrival to the ED, a RA recorded the current NEDOCS score for each patient, as well as demographic information and the location of the patient offload. The NEDOCS score was recorded from the last score calculated by the charge nurse, typically every 1–2 h throughout the day. The AOD was determined as the time that elapsed from ambulance arrival until both EMS report was given and movement of the patient off of the EMS stretcher was completed. Times were recorded by the RA from their own watch that was synchronized to the central clock in the ED at the beginning of each shift. NEDOCS scores were grouped according to the standard NEDOCS groupings for the scores over 100 (indicative of ED crowding) and the scores of 0–100 were included in one group. Data were entered into SPSS^®^ Statistics 19 (IBM^®^) and analyzed to determine the median AOD in minutes (min) overall, and for four groups of NEDOCS score ranges (group 1 = 0–100, group 2 = 101–140, group 3 = 141–180, group 4 ≥181). Patient race and gender were also evaluated for association with AOD.

## Results

The patient volume delivered to the study ED was 15,411. A convenience sample of 483 patients (3%) was observed. The AOD ranged from 0–157 min with a median of 11 min and interquartile range of 5–21 min. Results are summarized in Table 1. The AOD was determined to be dependent on a delay in EMS report to ED staff in 27.9% of cases and dependent on delay in availability of an ED stretcher in 72.1% of cases. The NEDOCS score ranged from 31 (normal) to 200 (disaster). There was a statistically significant difference between the NEDOCS score groups (*p* < 0.001). There was a statistically significant difference in AOD between groups 1 and 3 (*p* = 0.014) and between groups 2 and 3 (*p* = 0.011). There was no statistically significant difference in AOD when comparing patients of different race or gender.

**Table 1:** Ambulance offload delay times: total and by NEDOCS score group

	**Range**	**25**^**th **^**Percentile**	**Median**	**75**^**th **^**Percentile**	**% of total sample**
Total sample	0–157 min	5 min	11 min	21 min	100%
NEDOCS group 1 (0–100)	0–130 min	5 min	9.5 min	19 min	23.6%
NEDOCS group 2 (101–140)	0–85 min	5 min	10 min	18 min	41.3%
NEDOCS group 3 (141–180)	0–121 min	6 min	14.5 min	31.75 min	29.8%
NEDOCS group 4 (>180)	1–157 min	6 min	13.5 min	39.25 min	5.4%

Key outcomes are summarized above. Significant variability in AOD was noted across all NEDOCs groups, and therefore times are related as quartiles. There was a statistical difference between the NEDOCS score groups (*p* < 0.001).

### Current study limitations

The current study is limited to the academic level 1 trauma center in a city with five hospital destinations. The study hospital had both adult and pediatric EDs with separate staffing. The convenience sample represents only 3% of all patients arriving by EMS. The recording of the NEDOCS score at the study hospital was calculated based on the adult ED statistics, possibly leading to some loss in statistical accuracy of the association between the AOD and NEDOCS score. NEDOCS scores were recorded from the computer, as calculated by the ED charge nurse on a 1–2-h basis. If the RAs had calculated the NEDOCS score, there may have been a more accurate association between the score and the offload delay. No patients were excluded from the convenience sample, potentially leading to some skewing of the data because of patients arriving in cardiac arrest or with major trauma who would have been immediately seen. In order to address this issue, times have been reported as quartiles. In future study, the Emergency Severity Index (ESI), complaint type, and/or injury severity score could be used during data analysis to account for variations associated with patients with extremely high acuity presentations. If AOD data could be collected for patients delivered to all five hospitals, an evaluation of the entire system could be made.

## Discussion

When EMS crews are experiencing AOD they are not available to the EMS system. In addition to the concern for the safety of the public, the financial cost of readiness could also increase. When the cumulative AOD for an EMS system is significant, more EMS crews and ambulances may be required during a given time period in order to provide the same level of response to calls, thus leading to a lower unit hour utilization and increased cost to the system. In states where EMS personnel are not allowed to provide care after entering a hospital, AOD could represent a significant delay in patient care. In this study, delay in availability of an ED stretcher was the longer time interval contributing to AOD in over 70% of cases. Reduction in AOD in those cases may require increased capacity and/or improvements in hospital throughput, whereas reduction in AOD due to delay in EMS report may be more directly related to availability of nursing staff. It is important for administrators to understand the significance of AOD and for stake-holders to avoid confusing ambulance diversion with AOD. A number of papers have been published addressing strategies to reduce diversion hours [8-11]. These studies have shown the diversion hours can be reduced. However, these studies did not show convincing evidence of tangible benefit to the EDs or patients in the study systems. In a study by Asamoah et al., an EMS system seeking to reduce diversion hours addressed the issue by mandating reduction of the hours of diversion without making significant changes to the overall patient throughput. This led to an intended reduction of diversion time of 82%, but also an unintended increase in AOD of 32% [11]. In this study they found no improvement in ED crowding. A systematic review by Pham et al. found no clear evidence that ambulance diversion has any effect on morbidity or mortality [12]. As noted in the NAEMSP resource document, AOD likely represents a more serious risk to patient care than diversion [4]. This emphasizes the importance of AOD as a marker of efficiency of hospital throughput and the need for continued study.

## Conclusions

The median AOD was less than 30 min, and AOD was related to ED crowding. Offload delay in this study had a positive correlation with NEDOCS score. The median AOD was considered significant and raised concern as it may constitute a delay in patient care. This is especially true in the study location due to the fact that EMS personnel are not technically allowed to continue to care for patients after entering the hospital. Because the NEDOCS score appears to be predictive of AOD, it should be further investigated as a potential marker for EMS systems and could potentially be utilized during destination decision-making or as a tool for hospital EDs to initiate and terminate diversion status. Future study of the entire local five-hospital system will lead to greater understanding of the factors effecting AOD. Observation of AOD should be used to assess the effectiveness of future ED process improvement initiatives.

## Abbreviations

AOD: Ambulance offload delay; EMS: Emergency medical services; ED: Emergency department; EMTALA: Emergency Medical Treatment and Labor Act; NEDOCS: National Emergency Department Overcrowding Scale; RA: Research assistant.

## Competing interests

The authors declare that they have no competing interests. This work represents the results of a continuing quality improvement project. No funds or honoraria were received in the support of this study. No editorial or statistical assistance or rights were extended to or claimed by anyone other than the authors. No “ghost writing” occurred or was considered. In addition, all figures and tables are original work of the authors.

## Authors’ contributions

DRC designed the study and provided primary authorship. SW organized data collection efforts, provided statistical analysis, and provided editorial support services. NS assisted in initial study design and review of the manuscript. CV and KS provided data support and manuscript review services. All figures and tables are by DRC. All authors read and approved the final manuscript.

## Authors’ information

DRC is the Director of Prehospital Medicine and the Program Director for the EMS & Disaster Medicine Fellowship at SUNY Upstate Medical University. SW is an Associate Professor (PhD) and a member of the research faculty of the Department of Emergency Medicine at SUNY Upstate Medical University. NS is a Clinical Assistant Professor of Emergency Medicine at SUNY Upstate Medical University. CV and KS are medical students in the College of Medicine at SUNY Upstate Medical University.
